# Mechanism of action and therapeutic targeting of CD30 molecule in lymphomas

**DOI:** 10.3389/fonc.2023.1301437

**Published:** 2023-12-22

**Authors:** Zhaoxia Li, Wei Guo, Ou Bai

**Affiliations:** Department of Hematology, The First Hospital of Jilin University, Changchun, Jilin, China

**Keywords:** CD30, BV, lymphomas, mechanism of action, targeted therapy

## Abstract

At present, the treatment of lymphoma has entered the era of precision medicine, and CD30, as a transmembrane protein, has become an important marker to help the diagnosis and formulation of treatment plans for lymphomas. This protein is widely expressed in various types of lymphomas and can play a role through nuclear factor-κB (NF-κB), mitogen-activated protein kinase (MAPK), and other pathways, and ultimately lead to the up-regulation of CD30 expression to give tumor cells a survival advantage. Brentuximab vedotin (BV), as an antibody-drug conjugate (ADC) targeting CD30, is one of the first new drugs to significantly improve survival in patients with CD30+lymphomas. However, the biological function of CD30 has not been fully elucidated. Therefore, this review highlights the CD30-mediated tumor-promoting mechanisms and the molecular factors that regulate CD30 expression. We hope that a better understanding of CD30 biology will provide new insights into clinical treatment and improve the survival and quality of life of lymphoma patients.

## 1 Introduction

With the deepening of the research on the pathogenesis of lymphomas and the development of monoclonal antibody technology, antibody-drug conjugates (ADC) have emerged as novel and promising immunotherapy and have been approved for the treatment of multiple lymphoma subtypes. They usually consist of monoclonal antibodies connected to small molecule cytotoxic drugs through linkers, with high targeting and strong killing effects, which can interact with immune cells to exert anti-tumor immune effects, and at the same time interfere with the target function and inhibit signaling pathways, which in turn inhibit the tumor growth, to make the survival of lymphoma patients continuously improved (1). Among them, brentuximab vedotin (BV), which targets CD30, has shown high responsiveness and controllable toxicity and is an important advancement in lymphoma treatment.

BV, which consists of a monoclonal antibody targeting CD30 and the anti-tubulin agent monomethyl auristatin E (MMAE), coupled via a protease-sensitive linker, was first approved by the Food and Drug Administration (FDA) in 2011 for the treatment of relapsed/refractory classical Hodgkin’s lymphoma (R/R cHL) and systemic anaplastic large cell lymphoma (sALCL). Since then, it has been incorporated into the treatment of CD30+primary cutaneous anaplastic large cell lymphoma (pcALCL), peripheral T-cell lymphoma (PTCL), and mycosis fungoides (MF), etc., and has achieved significant efficacy (2–4).

CD30 is a member of the tumor necrosis factor receptor superfamily (TNFRSF), the expression of which is important for the diagnosis of a variety of lymphomas. For example, nearly 100% of tumor cells in patients with cHL and ALCL express CD30, making CD30 a diagnostic marker for these two types of lymphomas. Moreover, other lymphoma subtypes, including diffuse large B-cell lymphoma (DLBCL), primary mediastinal B-cell lymphoma (PMBL), MF, and many other subtypes of PTCL, express CD30 to varying degrees (5–7). In addition to the diagnosis and differentiation of lymphomas, CD30 expression is instructive in the prognostic evaluation of lymphomas. Some studies have shown that CD30 is a predictor of better survival in DLBCL patients (8), but in extranodal natural killer/T-cell lymphoma (ENKTL), the overall survival (OS) and progression-free survival (PFS) were significantly shorter in the CD30+group compared with the CD30-group (9).

In recent years, the in-depth study of CD30 and the mechanism of tumor development has laid the foundation for the treatment of lymphomas with CD30-targeting ADC drugs. This review will elaborate on the mechanism of CD30-mediated tumor promotion, explore the molecular factors that regulate CD30 expression and function, and search for more efficient potential therapeutic strategies for CD30+lymphomas based on BV.

## 2 Structure and biological function of CD30 protein

CD30, also known as Ki-1 antigen or TNFRSF8, is located on chromosome 1p36. In 1982, Karl Lennert’s team found that it could be recognized by the monoclonal antibody Ki-1, which can bind specifically to HL Reed-Sternberg (HRS) cells, so it was named the Ki-1 antigen (10). This molecule was subsequently identified as a 120kd transmembrane glycoprotein receptor belonging to the TNFRSF, TNFRSF8 (11). CD30 can be detected by immunohistochemistry (IHC), flow cytometry (FCM), enzyme-linked immunosorbent assay (ELISA), etc. CD30 is mainly expressed in a variety of immune cells, and the expression level of monocytes is higher. B cells, NK cells, dendritic cells (DC), T regulatory cells (Treg), activated CD4+and CD8+T cells, and eosinophils are also expressed, but not resting T cells (12, 13).

Structurally, CD30 has intracellular, trans-membrane, and extracellular domains. Its extracellular domain shares amino acid sequence homology with other TNFRSF members, containing six cysteine repeats. In inflammatory diseases and CD30+lymphomas, the extracellular portion of CD30 is easily cleaved by protein hydrolases into soluble fragments (sCD30) that are secreted into the plasma and detected by CD30 antibodies. The intracellular domain of CD30 has no homology to the other members but contains three sub-structural domains, D1, D2, and D3, that have independent functions. The D2 and D3 subdomains have binding sites for TNFR-associated factor (TRAF)-1, 2, 3, and 5, which mediate the activation of multiple signaling pathways, whereas the D1 subdomain does not need to bind to activate multiple signaling pathwaysv (6, 14–16). This conformation provides a structural basis for CD30 to fulfill its biological functions.

Given the importance of CD30 in lymphoma development, some scholars have tried to reveal the function of this protein through CD30 gene knockout mice at an early stage of the molecule’s emergence. Immature T cells need to undergo positive and negative selection in the thymus to fulfill their physiological roles, where positive selection is mainly responsible for inducing the differentiation of immature double-positive (DP) (CD4+CD8+) thymocytes expressing the T cell receptor (TCR) into mature single-positive thymocytes, whereas negative selection is responsible for the elimination of DP or single-positive thymocytes that have a high affinity for self-antigens (17). Amakawa et al. showed that the thymic volume of CD30-/-mice increased and the number of DP thymocytes increased, that is, in the absence of CD30, the negative selection of thymocytes was impaired, suggesting that CD30 plays a role in the negative selection of thymocytes. However, this study did not find that CD30 had significant effects on the proliferation and differentiation of memory T cells and the class-switched T-cell-dependent B cells (18). Gaspal et al. showed that CD30-/-mice were impaired in their ability to maintain follicular germinal center response and re-immune response, and when combined with OX40 ligand defects, the effect of CD30 on re-immune response was even more significant, suggesting that OX40 and CD 30 work together to regulate the proliferation and differentiation of memory T cells, which in turn promotes the occurrence of re-immune responses (19). These findings suggest that CD30 plays an important role in immune regulation and crosstalk between immune cells. In addition, with the further development of research, a large number of studies have found that CD30 can activate the nuclear factor-κB (NF-κB) and mitogen-activated protein kinase (MAPK) signaling pathways by recruiting TRAF factors, thus playing an anti-apoptotic and pro-survival role in tumor cells (20–22). CD30 has a variety of functions, depending on the target cell and its cell microenvironment. In the following, we will elaborate on the mechanism of CD30 in lymphoma to further uncover the mystery of the CD30 molecule.

## 3 CD30-Mediated tumor promotion mechanisms

### 3.1 CD30-Mediated signaling pathways and their association with lymphomas

The development of lymphoma is inextricably linked to the abnormal activation of multiple signaling pathways, including the NF-κB signaling pathway and the MAPK signaling pathway. The NF-κB family consists of five members, NF-κB1 (p105/p50), NF-κB2 (p100/p52), p65 (RELA), RELB, and REL, of which NF-κB1 (p105/p50), p65 (RELA), and REL are classical NF-κB pathway factors, and NF-κB2 (p100/p52)and RELB are non-classical pathway factors. They can form various homo-/heterodimers and are involved in a variety of biological processes such as cell proliferation and differentiation, and inflammatory responses (23). Several studies have demonstrated that HRS cells can express five NF-κB factors and have constitutive activation of the NF-κB pathway, which can reduce proliferation and increase apoptosis of HRS cells by inhibiting both classical and non-classical NF-κB pathways in HL cell line (24–28). Similarly, activation of the NF-κB signaling pathway has been reported in lymphomas such as DLBCL, mantle cell lymphoma (MCL), and PTCL (29–31). The MAPK pathway mainly involves four subfamilies, ERK, p38 MAPK, JNK, and ERK5, of which ERK and ERK5 mainly regulate cell growth and differentiation, and JNK and p38 MAPK play important roles in stress reactions such as inflammation and apoptosis (32). Joan Enric et al. performed gene expression and copy-number arrays analysis in 34 patients with plasmablastic lymphoma (PBL) and found that 49% of the cases had NRAS and KRAS mutations, which could lead to constitutive activation of the MAPK pathway (33). In addition, Mario I et al. detected the expression of p38 MAPK in 80 patients with DLBCL and found that p38 MAPK was highly expressed in tumor tissues (82%) and had poor sensitivity to the CHOP regimen, which was an independent risk factor affecting the prognosis of DLBCL (34). Overall, a large number of studies have verified that the activation of both signaling pathways is important for the survival of lymphoma cells.

**Figure 1:**
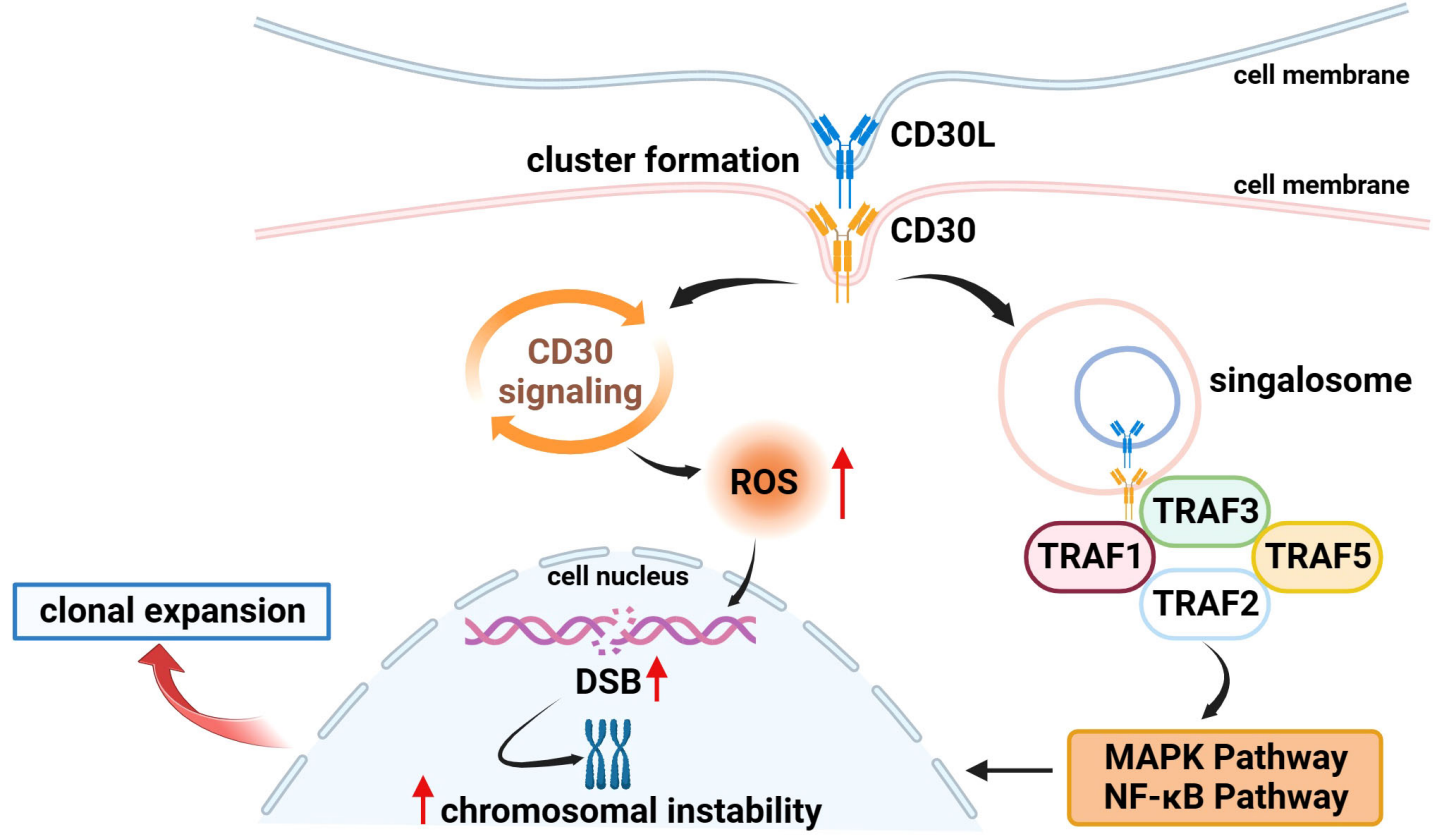
Association of CD30 with signaling pathways and genetics in lymphomas. (1) On the surface of cell membranes expressing CD30 and CD30L, CD30 and CD30L form clustered complexes and are internalized, while generating signal transducers that bind to TRAF-1, 2, 3, and 5, inducing the activation of the NF-κB and MAPK pathways, and participating in the processes of tumor cell proliferation and apoptosis. (2) CD30 signaling upregulates the level of ROS in tumor cells which in turn causes DSB, inducing chromosomal instability and clonal expansion.

Several studies have found that CD30 can activate NF-κB and MAPK signaling pathways through interaction with CD30 ligand (CD30L) or overexpression of CD30 itself, including the classical and non-classical pathways of NF-κB, ERK MAPK, JNK MAPK, and p38 MAPK (20, 30, 35–37). CD30 binds to the CD30L to form a trimer that recruits TRAF-1, 2, 3, and 5 to its intracellular domain, and TRAF members, as key signal transduction factors, transmit signals between surface receptors and transcriptional regulators, thereby inducing the activation of the NF-κB and MAPK pathways (38, 39) (**Figure 1**). ADCs can not only target and kill cancer cells through the release of small-molecule cytotoxic drugs, which is the core mechanism of action but also inhibit signaling pathways to suppress tumor growth (1). It has been demonstrated that trastuzumab, which is a second-generation ADC with BV, can bind to the HER2 receptor on the surface of cancer cells, blocking the formation of heterodimers between HER2 and HER1, HER3, or HER4, and then inhibiting the signaling pathways, such as PI3K or MAPK, to induce apoptosis (40). Therefore, we speculate that BV, as an ADC drug targeting CD30, can also play an anticancer role by specifically binding to CD30 on the surface of tumor cells and inhibiting the NF-κB and MAPK signaling pathways, but there are few relevant studies, and further basic research is needed to validate this in the future.

Moreover, Nakashima et al. found an interesting phenomenon that CD30 signaling is a more dynamic process mediated by trogocytosis. Trogocytosis is the process by which cells take up extracellular biomolecules, particulate matter, or fluids into the cell through the formation of vesicles by plasma membrane invagination to maintain normal cellular metabolic activities. For example, it has been shown that T cells can take up and internalize MHC molecules from antigen-presenting cells (APCs) via TCR or CD28 receptors (41, 42). Similar to this process, Nakashima et al. observed in adult T-cell leukemia/lymphoma (ATL) cells that CD30 and CD30L can form huge clusters on the membrane surface of cells expressing CD30 and CD30L and that CD30 triggers the internalization of the cluster complexes by extracting CD30L and part of its plasma membrane from neighboring cells, and these complexes simultaneously generate signal signalosomes that activate intracellular signaling and are ultimately degraded in lysosomes (43) (**Figure 1**).

### 3.2 Association of CD30 with the tumor microenvironment

The tumor microenvironment (TME) is a highly structured ecosystem composed of cellular components such as tumor cells, resident and recruited immune cells, and non-cellular components such as extracellular matrix, where immune cells contain various cell types such as T and B lymphocytes, tumor-infiltrating NK cells, and tumor-associated macrophages (TAMs), which together with their secreted factors, play an important role in tumorigenesis and not just a bystander role (44). For example, the crosstalk phenomenon between T follicular helper (Tfh) and follicular lymphoma B cells increases the levels of CCL17 and CCL22, induces the migration of Treg and CD4+T cells, and stimulates the release of more chemokines, resulting in the formation of immunosuppressive TME that promotes the survival and growth of tumors (45, 46). Tumor-infiltrating NK cells can release cytotoxic T cells (CTL) to kill tumor cells (47), while TAM can secrete transforming growth factor-β (TGF-β), which can not only directly inhibit the activation of NK cells, but also indirectly inhibit NK cells by inducing the differentiation of Treg cells (48, 49). In addition, there are a variety of cells, such as tumor-associated neutrophils (TANs), myeloid-derived suppressor cells (MDSCs), and cancer-associated fibroblasts (CAFs), which can participate in the regulation of various biological processes such as angiogenesis, matrix formation, and release of exosome through the expression of different biomarkers (50, 51). In conclusion, there is a very complex and interrelated crosstalk phenomenon between tumor cells and immune cells in TME, under the influence of which leads to immune escape from lymphomas.

**Figure 2:**
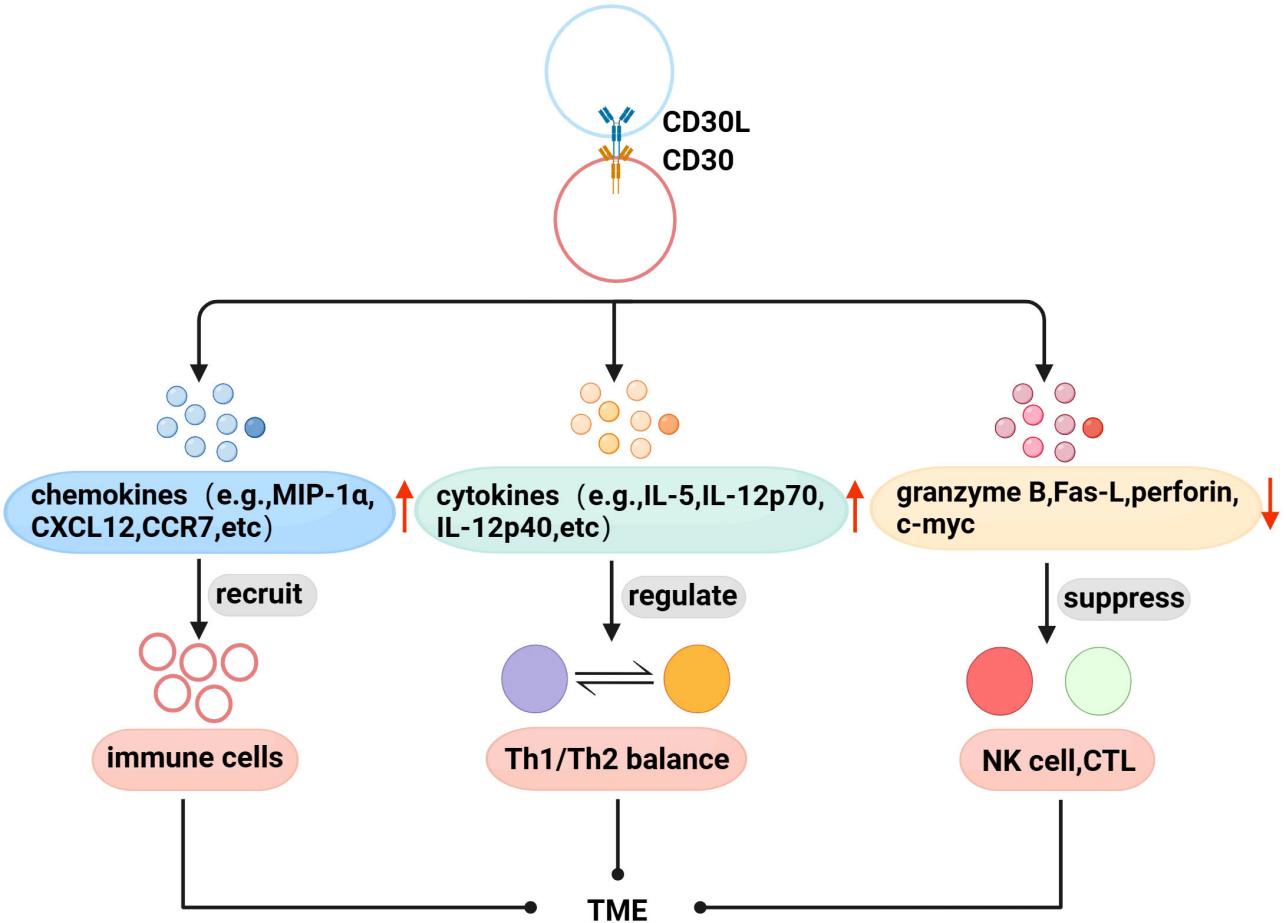
Association of CD30 with TME in lymphomas. (1) Promote the secretion of chemokines to recruit immune cells to the TME. (2) Upregulate the levels of Th1/Th2 balance-related cytokines to indirectly regulate the TME immune network. (3) Downregulate perforin, granzyme B, Fas-L, and c-myc to inhibit the proliferation and cytotoxic activity of NK cells, CTL in TME.

With further research, there is evidence that CD30 and TME are also linked. Immune cells are recruited to the TME to exert physiological effects through interactions between chemokines and their receptors. Fischer et al. found that mast cells exist in HL, and 66% of CD30L+cells are mast cells (52), and later found that cross-linking of CD30 with CD30L induced the secretion of the chemokines IL-8, macrophage inflammatory protein-1α (MIP-1α), and MIP-1β from mast cells (53). Vinante et al. found that the cross-linking of CD30 and CD30L upregulated the chemotactic activity of CXCL12 and the expression of its receptor CXCR4 in HL, and induced the release of CCL5 and CCL3 (54). These studies suggest that CD30 may be involved in the formation of TME by promoting the recruitment of immune cells. Th1/Th2 is in a relatively balanced state when it is normal, but when the body has functional abnormalities, the balance is often biased to one side, which is called “Th1/Th2 drift”,destroying the dynamic balance of the cytokine network in the body, leading to the generation and development of diseases (55, 56). Pellegrini et al. found that CD30 can upregulate the secretion of cytokines IFN-γ, IL-5, IL-4, IL-12 p70, and IL-12p40, and the interaction between these factors is the determining factor regulating Th1/Th2 balance (57). Therefore, CD30 can be used as an important molecule regulating the Th1/Th2 balance to coordinate the regulation of the immune network in TME. In addition, CD30 can also affect the proliferation and cytotoxic activity of immune cells in TME. NK and CTL cells can kill tumor target cells by releasing perforin, granzyme and Fas-L, etc. Muta et al. found that CD30 can downregulate the above molecules to directly inhibit the cytotoxic activity of NK and CTL, and downregulate c-myc to reduce the proliferation ability of CD30+CTL (58) (**Figure 2**).

**Figure 3:**
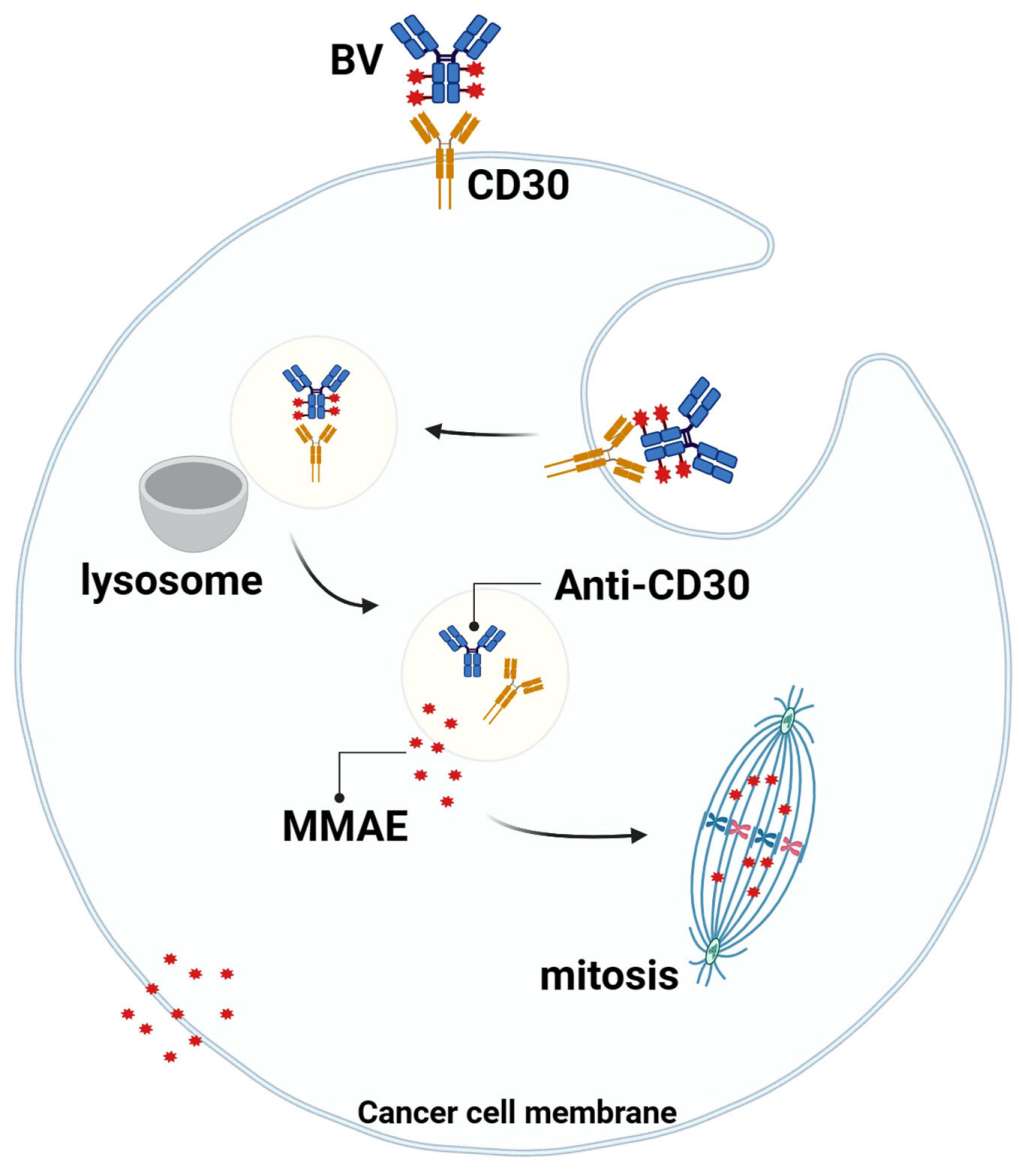
Mechanism of action of BV. BV binds to CD30 on the surface of tumor cells, the complex is endocytosed and transported to lysosomes, the linker is degraded by proteases, and MMAE is released into the cytoplasm, where it binds to microtubule proteins to arrest mitosis in the G2/M cell cycle and mediate apoptosis. At the same time, MMAE diffuses into the TME and exerts anti-tumor effects through the “bystander effect”.

Studies have shown that the MMAE portion of BV can be released into TME with the breakdown of CD30+lymphoma cells, and MMAE can freely cross the cell membrane, so it can play a bystander effect in TME for anti-tumor effects, which also explains why BV treatment is still effective when the expression rate of CD30 in lymphoma is low (59, 60) (**Figure 3**). Younes et al. showed that BV treatment resulted in regression of lymphoma masses and decreased levels of chemokines and inflammatory cytokines, suggesting that BV may interfere with TME by reducing cytokine release (61). In addition, Muller et al. found that BV can also induce DC cell maturation, upregulate the co-stimulatory molecules on the surface of T cells and B cells to promote cell activation, downregulate Treg levels, and increase the number of CD8 and CD4 tumor-infiltrating lymphocytes (TILs) in the early stage of BV treatment. This suggests that BV may ameliorate the imbalance between tumor-mediated immunosuppression and anti-tumor immunity in TME (62).

### 3.3 Association of CD30 with lymphoid genomic instability

In addition to the above mechanisms, existing studies have found that CD30 is also associated with the morphology and genetics of lymphoma cells to a certain extent. In 2018, Nakashima’s team reported that CD30 induced polylobation of human T-cell lymphotropic virus type 1 (HTLV-1)–infected cells by activating the PI3K pathway, and also observed that CD30 induced abnormal cell division and hyperploidy in HTLV-1–infected T cells, suggesting a biological link between CD30 and the active proliferation and chromosomal aberrations of HTLV-1–infected T cells (36). Subsequently, the team successively found that CD30 could cause DNA double-strand breaks (DSB) by upregulating the levels of intracellular reactive oxygen species (ROS) in ATL and cHL cells, thereby inducing chromosomal instability and clonal expansion (**Figure 1**), and with the progression of the disease, the amount of copy number loss of genes related to DSB repair would gradually increase (63, 64). Chromosome instability caused by mis-repaired DSB is one of the markers of human cancer development (65, 66). These studies confirm the association between CD30 and lymphoma genetics, which is a key factor in the progression of lymphoma, and future studies can be conducted to further explore the link between the two to explore the potential function of CD30 that is not yet known.

## 4 Mechanisms regulating CD30 expression

### 4.1 Gene regulation mechanisms of CD30

To explore the factors regulating CD30 expression, Croager et al. isolated and compared the promoter sequences of CD30 in humans and mice, and found that the two have many common and highly conserved transcription factor binding sequences, which may contain important elements regulating CD30 gene expression (67). Subsequently, three regulatory regions were found to play an important role in CD30 gene transcription: conserved Sp-1-binding sites, a downstream promoter element (DPE), and a microsatellite sequence (MS). The Sp1 site is a critical element required for initiating transcription of the CD30 gene, which recruits TATA-binding proteins that are normally involved in the formation of transcription initiation complexes around the TATA consensus sequence. The DPE is related to the transcriptional localization of the CD30 gene, and its mutations effect the position of transcription initiation. The MS can bind to proteins that inhibit the transcriptional activity of the CD30 promoter, which may be related to the dysregulation of CD30 expression in tumor cells (68). In addition, Watanabe et al. observed CpG islands containing 60 CpG dinucleotides in the promoter region, exons, and introns of the CD30 gene in HL and ALCL cell lines, and that methylation of the CpG islands repressed CD30 expression (69). A CpG island is a DNA segment of approximately 1000 base pairs, and most gene promoters are located within CpG islands, while DNA methylation refers to the selective addition of methyl groups to cytosine to form 5-methylcytosine in the presence of DNA methylation transferase, which regulates gene expression by recruiting proteins involved in gene repression or by inhibiting the binding of transcription factors to DNA, a process that can occur within CpG islands (70). This study suggests that the status of CpG islands within the CD30 gene can influence the transcriptional activity of the CD30 gene.

Super-enhancers (SEs) are regions composed of large clusters of active enhancers near the upstream and downstream region of gene promoters, which not only play key roles in the growth and development of healthy cells but also regulate key oncogenes, which are of great significance for tumorigenesis and characterization maintenance (71). Wong RWJ and Liang HC’s team conducted SE-associated gene analysis on ATL and ALCL cell lines respectively, and the results showed that CD30 gene locus existed in the SE region of both cell lines, suggesting that SEs may also play important roles in regulating CD30 expression (72, 73). In addition, YJ Huo et al. integrated genomic and transcriptomic data, divided CD30+DLBCL into three subtypes and screened out the corresponding three genes with the highest mutation frequency: *TNFAIP3*, *SOCS1*, and *CIITA*. Further validation analyses of the three genes on DLBCL cell lines revealed that the silencing of *TNFAIP3*, *SOCS1*, and *CIITA* genes could upregulate CD30 expression and make the DLBCL cell line sensitive to BV (74).

Several important transcription factors have also been found to regulate CD30 expression. STAT3 can directly up-regulate the expression of CD30 by binding to the CD30 promoter region in ALCL cell lines, and it can also bind to two highly conserved STAT3 sites in the CD30 intron, which indirectly suggests that they may be potential enhancer regions (75). Interferon regulatory factor-4 (IRF-4) can also bind to the CD30 promoter region to induce its transcriptional activity, and NF-κB subunit NF-κB2 (p100/p52)and RELB are transcriptional activators of IRF-4 in PTCL cell lines, suggesting a positive feedback loop between CD30, NF-κB, and IRF-4 (76). The AP-1 transcription factor subunit JUNB also binds to the CD30 promoter region and constitutively activated JUNB acts on the AP-1 site of CD30 to induce CD30 overexpression (77, 78). Liang HC et al. found that another AP-1 subunit, BATF3, can be recruited to the CD30 regulatory region, and that knockdown of BATF 3 decreased CD30 expression in ALCL cell lines, indicating that BATF 3 is also a key factor in regulating CD 30 expression (73).

### 4.2 Association between CD30 expression and viral infections

Recent studies have found that there is a synergistic effect between CD30 expression and viral infection. Hu S et al. demonstrated that EBV+DLBCL had a higher percentage of CD30 expression than EBV-DLBCL and that CD30+EBV+DLBCL had a lower survival rate than CD30+EBV-DLBCL patients (8). This conclusion was further validated by the findings of Ling L et al, who also applied gene set enrichment analysis (GSEA) in that study to confirm the enrichment of the JAK/STAT signaling pathway in EBV+DLBCL, which was consistent with the results of increased expression of phosphorylated STAT3 (pSTAT3) in EBV+DLBCL, suggesting that STAT3 may be activated by EBV (79). It has been shown that STAT3 can be activated by latent membrane protein 1 (LMP1) of EBV (80, 81), and EBV nuclear antigen 2 (EBNA2) is a transcriptional co-activator of STAT3 (82), so the results of this study by Ling L et al. may support the synergistic effect of LMP1 and EBNA2 on the activation of STAT3, and we can also further speculate that EBV may regulate CD30 expression through LMP1 and EBNA2 mediated STAT3 activation. Subsequently, Minamitani et al. confirmed that LMP1 could indeed upregulate CD30 expression by RNA sequencing (83), but the specific mechanism is still unclear. Ling L et al. also performed gene expression profiling (GEP) analysis on CD30+EBV+DLBCL and CD30+EBV-DLBCL in the above study and found that a total of 68 genes were differentially expressed between the two groups, which are involved in a variety of biological processes such as activation of signaling pathways and cell proliferation. For example, *RSF1* usually inhibits the NF-κB pathway, and its down-regulation in CD30+EBV+DLBCL may contribute to NF-κB activation. It has also been demonstrated that LMP1 of EBV activates the NF-κB pathway (84, 85). Together, this explains why DLBCL co-expressed with EBV and CD30 is characterized by enhanced NF-κB pathway activity, accelerated cell proliferation, and cell cycle progression. Although the application of BV in EBV+lymphoma is rarely reported, the above study gives us the insight that BV may become one of the potential combination therapeutic agents for patients with EBV+lymphoma with a deeper understanding of the mechanism of CD30’s role in EBV+lymphoma from the perspective of EBV.

## 5 Clinical studies of CD30-targeted therapy for lymphomas

### 5.1 Key clinical studies of BV for CD30+lymphomas

#### 5.1.1 HL

The phase III ECHELON-1 study conducted by Straus et al. showed that the 5-year PFS rate of BV in combination with AVD regimen was significantly higher than that of ABVD regimen for the treatment of untreated HL, and significant benefits from the combination regimen were also observed in the PET-CT-negative subgroup of patients, and the incidence of adverse events was similar in the two groups (86), which has established the first-line treatment status of BV combination chemotherapy in patients with untreated HL. In addition, Ansell et al. reported the results of an overall survival analysis of A+AVD as compared with ABVD from the ECHELON-1 trial, as well as long-term safety data, after approximately 6 years of follow-up. The results showed that the 6-year OS and PFS were higher in the A+AVD group than in the ABVD group and that fewer patients in the A+AVD group received subsequent therapy (including transplantation) and developed second cancers than in the ABVD group (87). A single-arm phase IV study conducted by Walewski et al. showed that in patients with R/R HL who were not candidates for hematopoietic stem cell transplantation (HSCT) or multiagent chemotherapy, BV monotherapy resulted in an overall response rate (ORR) of 50%, with a median PFS and OS of 4.8 months and unmet, respectively, and ultimately HSCT was feasible for 47% of the patients, suggesting that BV monotherapy has shown anti-tumor effects in salvage therapy for R/R HL patients (88). In addition, Advani et al. found for the first time that BV combined with nivolumab is also a safe and effective salvage treatment for R/R HL, with ORR rates, 3-year PFS rates and OS rates as high as 85%, 77% and 93%, and patients undergoing consolidation with allogeneic HSCT (allo-HSCT) are estimated to have 3-year PFS rates of 91% (89). In addition, the AETHERA study found that BV maintenance therapy was also able to provide sustained benefit to patients after autologous HSCT (ASCT), and the 5-year PFS rate in the BV group was significantly higher than that in the placebo group (90).

#### 5.1.2 PTCL

The phase III ECHELON-2 study conducted by Horwitz et al. showed that the median PFS time of BV combined with the CHP regimen for CD30+PTCL was significantly prolonged compared with the CHOP regimen, and the combination regimen had a consistent advantage in all subgroups (3), which has thus established BV combined with chemotherapy as the first-line treatment in CD30+PTCL. The phase II study conducted by Pro et al. showed that the ORR of BV monotherapy for R/R sALCL was 86%, the 5-year PFS rate and OS rate were 39% and 60%, respectively, and the median PFS and OS of patients receiving consolidation with allogeneic HSCT did not reach (91), and this study confirmed the status of BV monotherapy in R/R sALCL. In addition, the phase III ALCANZA study confirmed that BV monotherapy provides a survival benefit for previously treated patients with CD30+MF and pcALCL, and the ORR and median PFS in the BV group were significantly higher than those in the conventional treatment group (92).

#### 5.1.3 B cell lymphomas

A single-arm phase I/II study by Svoboda et al. explored the efficacy and safety of the BV-R-CHP regimen for the first-line treatment of CD30+B cell lymphomas, and the results showed that the ORR was 100%, and none of the five DLBCL patients progressed until the publication of the study (93). Another open phase II study evaluated the efficacy of BV in R/R CD30+NHL and found that BV was effective in R/R DLBCL with varying levels of CD30 expression (94). Moreover, a phase I/dose extension study by Ward et al. suggested that BV in combination with lenalidomide was also a potential treatment option for R/R DLBCL (95).

The emergence of BV has provided new therapeutic options for patients with lymphomas, and a number of clinical studies containing BV regimens for the treatment of HL, PTCL, and B cell lymphomas are still ongoing, including the role of BV in combination with chemotherapy, BV in combination with immune checkpoint inhibitors, and post-transplantation BV maintenance (**Tables 1**–**3**).

**Table 1:** Ongoing clinical studies of BV in the treatment of HL.

NCT number	Stage	Intervention plan	Disease type	Primary endpoints
NCT05675410	III	BV+Nivolumab vs.ABVD/AVD/BEACOPP	Untreated cHL	PFS
NCT05595447	II/III	Induction : BV+Nivolumab/Pembrolizumab;Consolidation : ASCT;Maintenance : BV+Nivolumab/Pembrolizumab	R/R HL	PFS
NCT05243693	II	BV-DHAP	R/R cHL	CRR
NCT05180097	II	BV+Pembrolizumab vs.GDP	R/R cHL	CRR
NCT05100056	IV	Observational	HL with ASCT	PFS
NCT05039073	II	BV+Nivolumab	R/R cHL	ORR
NCT04685616	III	A2VD vs.ABVD	Untreated cHL	PFS
NCT04561206	II	BV+Nivolumab	R/R cHL	PFS
NCT04378647	II	Induction : BV ± ESHAP; Maintenance : BV	R/R cHL	CRR
NCT03907488	III	BV-AVD vs.Nivolumab-AVD	Untreated cHL	PFS
NCT03646123	II	Part A:A-AVD; Part B and C:AN-AD	Untreated cHL	Part A:Safety;Part B and C:CRR
NCT03576378	I/II	BrEPEM-LH-22017	Untreated cHL	Phase I:MTD;Phase II : CRR,Safety
NCT03474133	II	BV	R/R cHL	CCR
NCT03233347	II	Induction:A-AVD; Maintenance : Nivolumab ± BV	Untreated cHL	PFS
NCT02927769	II	BV+Nivolumab,BV+Bendamustine	R/R cHL	EFS,CMR
NCT02661503	III	BrECADD vs.BEACOPP	Untreated cHL	PFS

ABVD, Adriamycin, Bleomycin, Vinblastine, Dacarbazine; AVD, Adriamycin, Vinblastine, Dacarbazin; BEACOPP, Bleomycin, Etoposide, Adriamycin, Cyclophosphamide, Vincristine, Procarbazine, Prednisone; DHAP, Dexamethasone, Cisplatin, Cytarabine; GDP, Gemcitabine, Cisplatin, Dexamethasone; A2VD, Adriamycin, BV, Vinblastine, Dacarbazine; ESHAP, Etoposide, Methylprednisolone, Cytarabine, Cisplatin; A-AVD, BV plus Adriamycin, Vinblastine, Dacarbazine; AN-AD, BV, Nivolumab plus Adriamycin, Dacarbazine; BrEPEM-LH-22017, BV, Cyclophosphamide, Procarbazine, Prednisone, Etoposide, Mitoxantrone; BrECADD, BV, Etoposide, Adriamycin, Cyclophosphamide, Dacarbazine, Dexamethasone; CRR, Complete response rate; CCR, Continuous complete response; MTD, Maximum tolerated dose; EFS, Event free survival; CMR, Complete metabolic response.

**Table 2:** Ongoing clinical studies of BV in the treatment of PTCL.

NCT number	Stage	Intervention plan	Disease type	Primary endpoints
NCT05673785	II	BV-CHP	Untreated PTCL	ORR, Safety
NCT05442554	IV	BV	R/R MF, pcALCL	ORR
NCT05414500	I	BV+Mogamulizumab	R/R MF, SS	Safety
NCT05357794	II	BV+Ultra-Low-Dose Total-Skin Electron Beam	Untreated and R/R MF, SS	ORR
NCT05313243	II	BV+Pembrolizumab	R/R PTCL, CTCL	BOR
NCT04998331	IV	Observational	R/R CD30+lymphomas	ORR, Safety
NCT04837222	IV	Observational	Untreated and R/R CD30+lymphomas	Safety
NCT04795869	II	BV+Pembrolizumab	R/R PTCL	ORR
NCT04569032	II	BV-CHP	Untreated PTCL(CD30 expression<10%)	ORR
NCT03587844	II	BV	Untreated MF, LyP, SS	ORR
NCT03409432	II	BV+Lenalidomide	R/R PTCL	ORR
NCT03264131	II	BV-CHEP	Untreated and treated (up to 1 course of chemotherapy) ATL	CRR
NCT03246750	I/II	B-MAD	Untreated ENKTL	Safety
NCT03217643	II	BV-CHP	Untreated EATL Type 1	PFS
NCT03187210	I/II	BV-BeEAM vs.BeEAM	CD30+lymphomas	Phase I: MTD;Phase II: DFS
NCT02616965	I	BV+Romidepsin	Untreated and R/R CTCL	MTD, DLT
NCT02588651	II	BV	R/R PTCL(CD30 expression<10%)	ORR
NCT01716806	II	BV	Untreated PTCL	ORR
NCT01352520	II	BV	R/R ALCL, MF, LyP	ORR

LyP, Lymphomatoid papulosis; SS, Sézary syndrome; CTCL, Cutaneous T-cell lymphoma; EATL, Enteropathy-associated T-cell lymphoma; CHP, Cyclophosphamide, Adriamycin, Prednisone; CHEP, Cyclophosphamide, Adriamycin, Etoposide, Prednisone; B-MAD, BV plus Methotrexate, L-Asparaginase, Dexamethasone; BeEAM, Bendamustine, Etoposide, Cytarabine, Melphalan; BOR, Best overall response; DFS, Disease-free survival; DLT, Dose limited toxicity.

**Table 3:** Ongoing clinical studies of BV in the treatment of B cell lymphomas.

NCT number	Stage	Intervention plan	Disease type	Primary endpoints
NCT04745949	II	BV+R-CHP+Nivolumab	Untreated PMBCL	CRR
NCT04587687	II	BV+Bendamustine	R/R FL	CRR, BOR
NCT04404283	III	BV, Placebo+R2	R/R DLBCL	PFS
NCT03356054	I/II	BV-R-DHAP	R/R DLBCL	Phase I:MTD;Phase II:CRR

FL, Follicular lymphoma; PMBCL, Primary mediastinal large B cell lymphoma; R-CHP, Rituximab plus Cyclophosphamide, Adriamycin, Prednisone; R2, Lenalidomide, Rituximab; R-DHAP, Rituximab plus Dexamethasone, Cisplatin, Cytarabine.

### 5.2 Novel CD30-targeted therapeutic agents

The successful treatment experience of BV has laid the foundation for the development of novel anti-CD30 therapies, which mainly include chimeric antigen receptor T (CAR-T) cells and bispecific antibodies. A phase I study showed that the ORR of CD30 CAR-T therapy for R/R cHL patients was 33% (96), and a follow-up study showed that CD30 CAR-T therapy in combination with chemotherapy further improved the clinical outcome of patients with R/R cHL, with an ORR of 62% and 1-year PFS and OS of 36% and 94%, respectively (97). The bispecific antibody AFM13 (CD30/CD16A) is an innovative intrinsic immune cell agonist, which activates innate immunity by specifically binding to CD30 on the surface of tumor cells and CD16A on the surface of intrinsic immune cells such as NK cells to exert antibody-dependent cell-mediated cytotoxicity (ADCC) (98, 99). A phase I/dose extension study showed an overall disease control rate of 61.5% (PR 11.5%, SD 50%) for AFM13 in patients with R/R HL (98), and a phase Ib study also showed an anti-tumor effect of AFM13 in combination with pembrolizumab in patients with R/R cHL, which the ORR is as high as 83% (99). In addition, based on the fact that both CD30 and CD137 (4-1BB) are expressed on HRS cells and that approximately 86% of tumor cells from cHL patients express CD137, Rajendran et al. developed a CD30/CD137 bispecific antibody, which was validated to show good specificity and ADCC (100). However, the efficacy and safety of the above regimens remain to be verified in high-quality, large-sample RCT studies.

### 5.3 Potential molecular strategies to improve the efficiency of CD30 targeting

Extracellular vesicles (EVs) are bilayer lipid vesicles secreted by cells or microorganisms with diameters of 100-1000 nm, containing rich in proteins, nucleic acids, and other molecules, which can act on target cells through proximal or distant intercellular communication, thus affecting the gene expression and biological functions of target cells (101). In recent years, it has been found that BV therapy is still effective even if the expression rate of CD30 in lymphomas is low, and Lobastova et al. proposed that CD30+EV may play an important role in this process. This study found that CD30+EV could bind not only to DLBCL cell lines but also to fluorescein-labeled BV, creating conditions for BV to kill CD30-tumor cells, and both confocal microscopy and FCM showed that the binding and uptake of BV depended on CD30+EV, so only in the presence of CD30+EV can BV kill CD30-tumor cells (102). However, this cross effect is limited by the cleavage of CD30 by the A Disintegrin And Metalloproteinase 10 (ADAM10). EVs not only carry CD30 molecules but also contain ADAM10, and the sCD30 formed after cleavage can no longer assist BVs in their anti-tumor effects (103, 104). Therefore, inhibiting the cleavage of CD30 by ADAM10 might improve the targeting efficiency of BV. Matthey et al. found for the first time that the application of hydroxamate inhibitors of metalloproteinases (BB-3644) can significantly reduce the sCD30 concentration, prolong the survival time of cHL xenografted mice, and improve the survival rate of mice (105). However, the results of a phase I/dose-expansion study by Wall et al. showed significant musculoskeletal toxicity in HL patients treated with BB-3644, thus terminating the clinical development of the drug (106). Since then, some studies have found more specific ADAM10 inhibitors, such as LT4 and CAM29, MN8, and ADAM10 antibody 8C7, which have fewer side effects and may become candidate drugs for combination with BV to improve the targeting efficacy of BV (107–109), which is worthy of further research to evaluate in the future.

## 6 Conclusion

CD30 is widely expressed in various types of lymphomas, and clinical studies in the last decade have demonstrated that this molecule is a high-value therapeutic target for lymphomas, and utilization of this target can improve the prognosis of patients with different CD30+lymphomas. Although existing studies have found that CD30 has rich biological functions that allow it to have multiple effects in tumor cells, such as anti-apoptosis and promotion of survival, its biological and clinical significance has not yet been fully elucidated, and remains to be further explored. As an ADC drug targeting CD30, BV has shown reliable therapeutic effect and good safety in both first-line treatment and salvage treatment, bringing new hope to lymphoma patients. However, more large-scale clinical trial data are needed in the future to continue to optimize the clinical application of BV, in order to bring greater clinical benefits to lymphoma patients.

## Author contributions

ZL: Writing – original draft. WG: Writing – review & editing. OB: Writing – review & editing.
